# A Sexually Conditioned Switch of Chemosensory Behavior in *C. elegans*

**DOI:** 10.1371/journal.pone.0068676

**Published:** 2013-07-04

**Authors:** Naoko Sakai, Ryo Iwata, Saori Yokoi, Rebecca A. Butcher, Jon Clardy, Masahiro Tomioka, Yuichi Iino

**Affiliations:** 1 Department of Biophysics and Biochemistry, Graduate School of Science, The University of Tokyo, Bunkyo-ku, Tokyo, Japan; 2 Department of Biological Chemistry and Molecular Pharmacology, Harvard Medical School, Boston, Massachusetts, United States of America; Harvard University, United States of America

## Abstract

In sexually reproducing animals, mating is essential for transmitting genetic information to the next generation and therefore animals have evolved mechanisms for optimizing the chance of successful mate location. In the soil nematode *C. elegans*, males approach hermaphrodites via the ascaroside pheromones, recognize hermaphrodites when their tails contact the hermaphrodites' body, and eventually mate with them. These processes are mediated by sensory signals specialized for sexual communication, but other mechanisms may also be used to optimize mate location. Here we describe associative learning whereby males use sodium chloride as a cue for hermaphrodite location. Both males and hermaphrodites normally avoid sodium chloride after associative conditioning with salt and starvation. However, we found that males become attracted to sodium chloride after conditioning with salt and starvation if hermaphrodites are present during conditioning. For this conditioning, which we call sexual conditioning, hermaphrodites are detected by males through pheromonal signaling and additional cue(s). Sex transformation experiments suggest that neuronal sex of males is essential for sexual conditioning. Altogether, these results suggest that *C. elegans* males integrate environmental, internal and social signals to determine the optimal strategy for mate location.

## Introduction

Animals possess a unique array of sensory abilities to locate their mating partners. Many organisms show innate behaviors in which they are attracted to reproductive partners, often relying on chemical signals that carry information of species and sexual identity. In addition to such primary sensory mechanisms, diverse sensory modalities can be additionally recruited in a process called sexual conditioning [1]. Sexual conditioning is a form of classical conditioning where the presence of mating partners is associated with diverse environmental features, such as visual, olfactory or auditory information, which are memorized and thereafter serve as a useful secondary cue to locate mating partners [2]–[5]. Although the combination of the innate and sexually conditioned behaviors seems essential to seek for mating opportunities, little is known about the coordination and interplay between these two mechanisms.

Sensory mechanisms for finding reproductive partners have been well described in several animal models [6]–[8]. For example, the soil nematode *Caenorhabditis elegans* is an androdioecious species consisting of two sexes: hermaphrodites, which can reproduce by themselves through self-fertilization, and males, which produce only sperm and must mate with hermaphrodites to produce offspring (www.wormatlas.org). Two sensory mechanisms for finding mating partners are known in *C. elegans* males [6]. First, males are attracted to hermaphrodite pheromones [9]–[11], the chemical nature of which is a mixture of sugar derivatives called ascarosides as well as other non-ascaroside compounds [12]–[14]. The pheromones are received by a few pairs of head sensory neurons in the male [10], [11]. Second, males directly recognize hermaphrodites by contacting the body surface of hermaphrodites with their tails [15]. This event prevents dispersal behavior, keeping males close to hermaphrodites [16], [17]. Thus mate-search behavior seems to be controlled by a limited set of sensory functions in the male.

In contrast with the mate-search behavior, diverse sensory modalities are used to explore bacterial food in *C. elegans* [18]. For example, various food-related odors and salts attract worms [19], [20]; they also migrate to temperature and oxygen concentration that they have experienced during feeding [21], [22]. In addition, experience of starvation reverses the direction of these sensory responses: worms avoid odors, salts, temperature and oxygen concentration that they have experienced during starvation 18,22. To date, the food availability-dependent plasticity of sensory response has been studied almost exclusively in hermaphrodites, which are easier to propagate due to self-fertilization. Unlike hermaphrodites, however, males absolutely require mating partners to reproduce, just like they need bacterial food to survive, raising a possibility that the sensory mechanisms used for food search may also be used for mate-search behaviors in the male.

In this report, we studied plasticity of gustatory behavior in the male. Sodium chloride is a chemoattractive taste for worms fed on bacterial lawn, but it becomes chemorepulsive after experiencing starvation in the presence of the salt [23]. We show that males are attracted to sodium chloride after starvation, rather than avoiding it, when they are kept with hermaphrodites, suggesting that the presence of mating partners overrides the aversive conditions of starvation. This effect, which we call sexual conditioning here, operates in unidirectional manner since hermaphrodite behavior is not affected by the presence of males. We further show that sexual conditioning requires at least two sensory functions for mate recognition: pheromone sensation and another cue that is specific to hermaphrodites. Our results reveal a secondary role of these mate-recognition systems in incorporating an additional sensory modality, namely salt taste, which is later used for optimizing the mate-search program of the male.

## Materials and Methods

### Strains and Culture

*C. elegans* strains were cultivated mainly on bacterial lawns of NA22 and maintained at 20°C using standard methods [24], because this bacterial strain has been used in our previous studies on salt/starvation learning. Note that sexual conditioning can be also observed for animals raised on OP50, the standard food source for *C. elegans* research (fig.S2). Strains used were wild-type Bristol N2, DR476 *daf-22(m130)* II, *him-5(e1490)* V, *mab-3(e1240)* II; *him-8(e1489)* X, CB3299 *mab-5(e1239)* III;*him-5(e1490) dpy-21(e428)* V, CB1309 *lin-2(e1309)* X, *odr-7(ky4)* X, *ceh-30(tm272)* X, and *odr-7(ky4)* X; *ceh-30(tm272)* X.

### Behavioral Assays

Assays of chemotaxis to NaCl was performed on a gradient of NaCl formed on an assay plate [6 cm diameter Petri dish poured with 3.5 mL of 2% agar, 5 mM potassium phosphate (pH 6.0), 1 mM CaCl_2_, 1 mM MgSO_4_] by placing a 0.13 mL agar plug containing 50 mM NaCl at 1.8 cm from the center of the plate 18 to 24 h before placing the animals. To clearly visualize the trace of worms, the agar surface was dried for 2 h just before placing the animals by opening the plate lid. To examine plasticity of chemotaxis to NaCl, washed animals were incubated on a conditioning plate [3.5 cm diameter Petri dish poured with 3.5 mL of 3% agar, 5 mM potassium phosphate (pH 6.0), 1 mM CaCl_2_, 1 mM MgSO_4_] with (NaCl-conditioned) or without (mock-conditioned) 50 mM NaCl for 2 h. Males were isolated from hermaphrodites 1 day before the behavioral assays. The numbers of males and hermaphrodites that were subjected to conditioning were 15-30 and 200, respectively, unless otherwise noted. After conditioning, single worms were placed at the center of the assay plate and allowed to run for 20 min. The chemotaxis score of a single animal was calculated as the sum of scores of the sectors through which the animal had traveled(fig.S1A). This scoring method was expected to maximize the information that can be drawn from the behavior of individual animals. Animals that failed to move from the starting point were excluded from the data set. The averages of chemotaxis scores were shown in each graph. Comparison between groups was performed using a Wilcoxon rank sum test. Crude pheromone preparation was generated as described [25], except that worms were cultured at 22.5°C for 16 d. This pheromone preparation was diluted in the indicated concentrations with 100 µl of water and spread over a conditioning plate immediately before conditioning started. Assays for pheromone attraction were performed as previously described with minor modifications [11]. Briefly, assay plates (3.5 cm diameter Petri dish) were uniformly covered with the *E. coli* strain NA22. Two microliters of diluted pheromones and water were each spotted within one of two circles of 5 mm diameter, which are defined as a scoring region and a control region, respectively (fig. S1B). During five minutes after the time when five animals were placed on the plate, their positions were tracked with a video-recording system for 15 minutes. Time in scoring region was defined as the time during which an animal continuously kept its position within the region. These data were statistically compared by Tukey’s test.

### Plasmid Construction and Germ-line Transformation

The *tra-1(gf)* and *fem-3(+)* expression vectors were constructed by using the GATEWAY system (Invitrogen). Details of the use of the GATEWAY system are available at our website: http://molecular-ethology.biochem.s.u-tokyo.ac.jp/ Gateway/Gateway_overview1.html. The *tra-1gf* and *fem-3(+)* cDNAs were obtained by PCR from *C. elegans* cDNA generated by reverse-transcription reaction using total RNA as a template and SuperScriptIII kit (Invitrogen) and ligated between KpnI and EcoRV in the pPD-DEST vector [pDEST-*tra-1gf* and pDEST-*fem-3(+)*]. The pENTR plasmids with promoter sequences contain the following regions of *C. elegans* genomic DNA: 2 kb of the *gcy-5* promoter, 1.2 kb of the *gcy-7* promoter, and 2.5 kb of *tag-168* promoter (*H20* promoter). Injection markers, *srj-54p::venus* and *ida-1p::mCherry*, were similarly generated from the pDEST plasmids for *mCherry* and *venus* and the pENTR plasmids containing 1 kb of the *srj-54* promoter and 2.5 kb of the *ida-1* promoter. Details of the constructs will be provided upon request.

Germ-line transformations were performed by standard microinjection methods [26]. Expression constructs for *tra-1(gf)* and *fem-3(+)* were injected at 20 ng/µl along with *srj-54p::venus* and *ida-1p::mCherry* as transformation markers at 20 ng/µl for each marker and pPD49.26 as a carrier DNA. The final concentration of injected DNA was 100 ng/µl. The expression of *srj-54p::venus* in AIM was used as a marker for neural sex [27].

## Results

### *C. elegans* Males Associate Salt Taste with the Presence of Hermaphrodites

**Figure 1:**
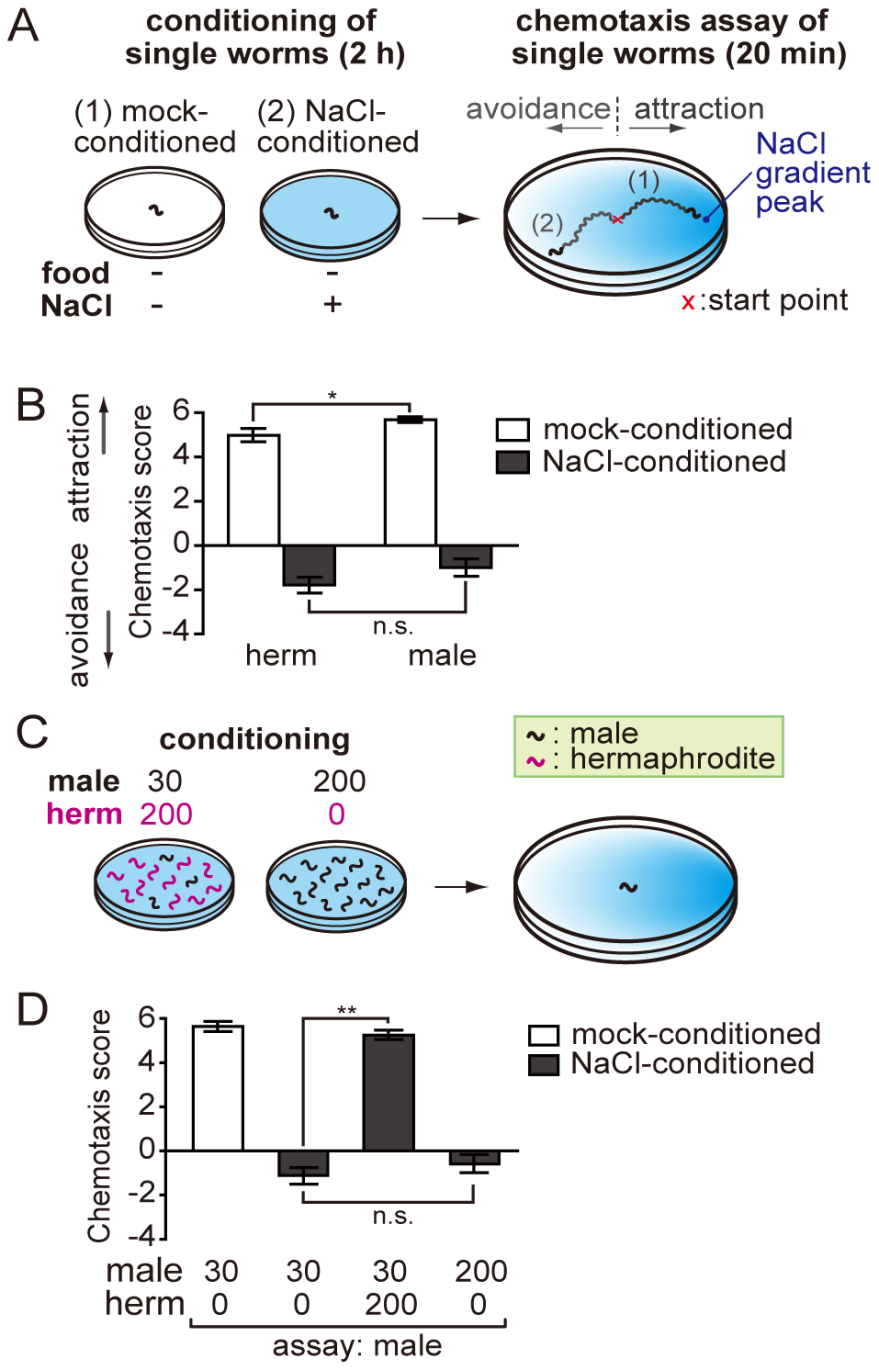
Sexual conditioning of salt chemotaxis behavior in the male. (A) Schematic of the assay for single worms in starvation-dependent plasticity of chemotaxis. Each worm was separately conditioned in the absence (1) or presence (2) of NaCl and tested for salt chemotaxis. Representative traces of (1) attraction to and (2) repulsion from the salt-concentration peak are shown. (B) Plasticity of salt chemotaxis in hermaphrodites (herm) and males. Single animals were subjected to starvation conditioning in the presence (NaCl-conditioned) or absence (mock-conditioned) of salt. No significant difference between males and females was observed in this paradigm. (C) Procedures to examine inter-sexual and inter-individual effects during salt/starvation conditioning of males. (D) Males are sexually conditioned to be attracted to salt after conditioning with hermaphrodites (herm), and this effect is not observed when conditioned with males. **, *P*<0.001; *, *P*<0.01; n.s., not significant. Error bars represent SEM. n = 55–80 animals for each condition.

A previous study examined sex difference in the plasticity of gustatory behavior and observed that males exhibit poor repulsion from salt after salt/starvation conditioning, concluding that males have lower ability of learning than hermaphrodites [28]. We presumed that the apparently lower ability of learning in males might be due to inter-individual interactions, and examined the reported sex difference under conditions that exclude interaction between individuals. We subjected males and hermaphrodites to salt/starvation conditioning and subsequent salt chemotaxis assays individually (Fig. 1*A*). To evaluate the behavior of each animal, we traced the tracks of the animals left on the agar surface. Then, each animal was given a score based on the sum of scores of the sectors through which the animal had traveled. Contrary to what was reported before [28], we did not observe a significant difference between males and hermaphrodites under these conditions (Fig. 1*B*), indicating that the two sexes have comparable learning abilities.

**Figure 2:**
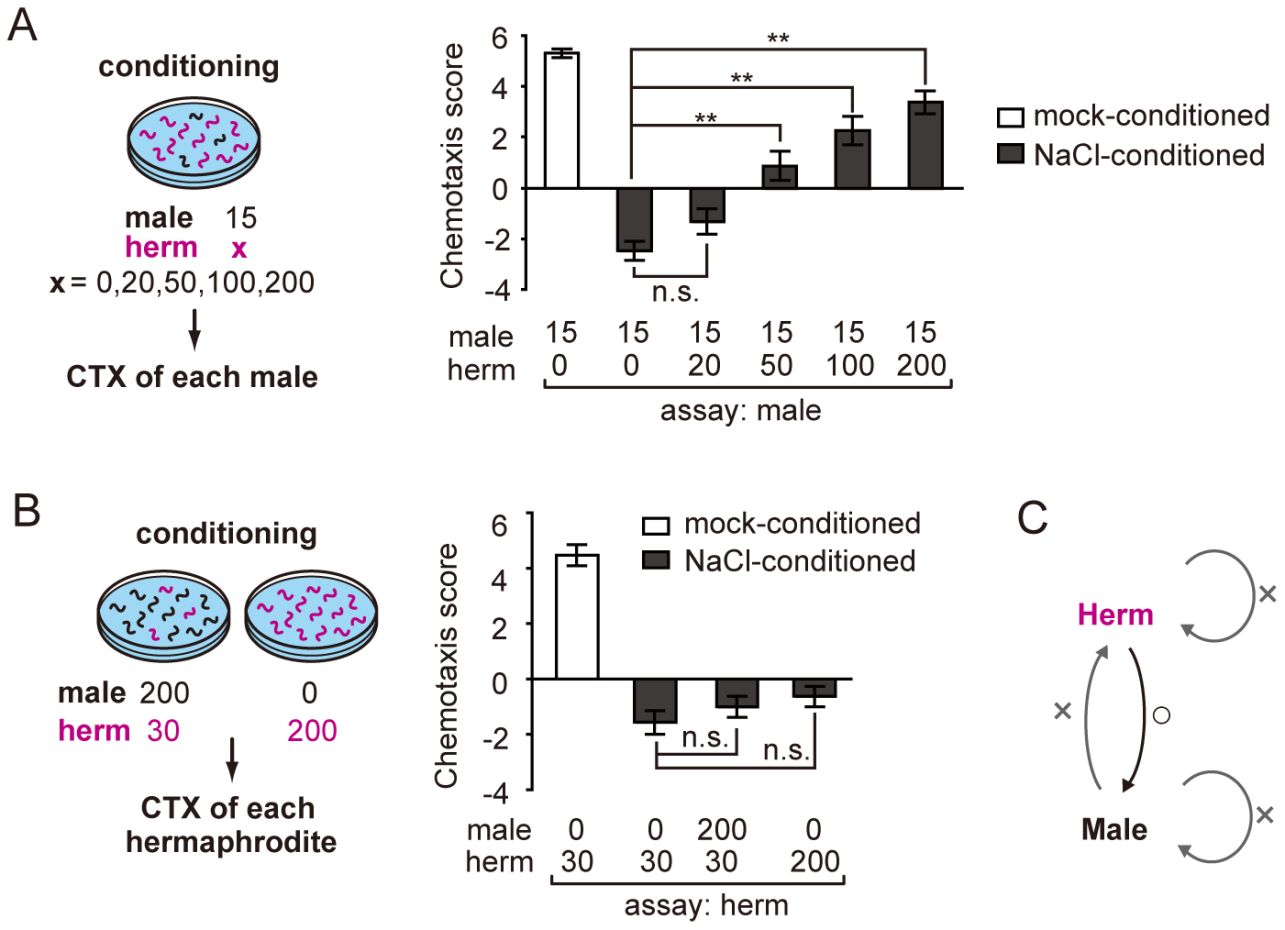
Hermaphrodite density is critical for sexual conditioning. (A) The dependency of sexual conditioning on the number of hermaphrodites present during conditioning. Salt attraction of males increases with increasing density of hermaphrodites during conditioning. CTX, chemotaxis. (B) The plasticity of salt chemotaxis in hermaphrodites is not significantly affected by the presence of males or hermaphrodites during salt/starvation conditioning. (C) Summary of inter-individual interactions that lead to suppression of salt aversion in males and hermaphrodites. **, *P*<0.001; n.s., not significant. Error bars represent SEM. n = 49–80 animals for each condition.

Because in the previous study the behavioral assays were performed using a population of worms including both sexes [28], we reasoned that the sex effect on learning might be due to inter-sex interactions between individual worms. To test this idea, male worms were mixed with a large number of hermaphrodites during conditioning and subsequently males were subjected to chemotaxis assays individually(Fig. 1*C*). As suspected, males showed attraction to salt instead of repulsion, suggesting that aversive learning is suppressed in males in the presence of hermaphrodites during salt/starvation conditioning (Fig. 1*D*). We also observed that more than a hundred hermaphrodites are required to fully evoke salt attraction, indicating that the hermaphrodite density is critical for this effect (Fig. 2*A*). In contrast to the hermaphrodite-to-male effect, males were not affected by the presence of a comparable number of males (Fig. 1*D*), suggesting that hermaphrodites have a sex-specific ability to elicit salt attraction in males. Next we turned to the hermaphrodite behavior and examined the interactions between individuals. We observed little or no effects on hermaphrodite behavior when they are conditioned either in the presence of a large number of males or hermaphrodites (Fig. 2*B*). Taken together, these observations suggest that males receive signals from hermaphrodites, which we call “sexual signals”, and that these signals lead to the suppression of aversive learning and the promotion of salt-attraction behavior, in a process we call “sexual conditioning”. The effect of sexual signals is similar to the presence of food, but the sexual signals are sex-specific and unidirectional (Fig. 2*C*). When salt is associated with the presence of mates by this mechanism, salt becomes a useful cue for males to locate and approach hermaphrodites.

### Sexual Conditioning is Mediated by Pheromonal Signaling

**Figure 3:**
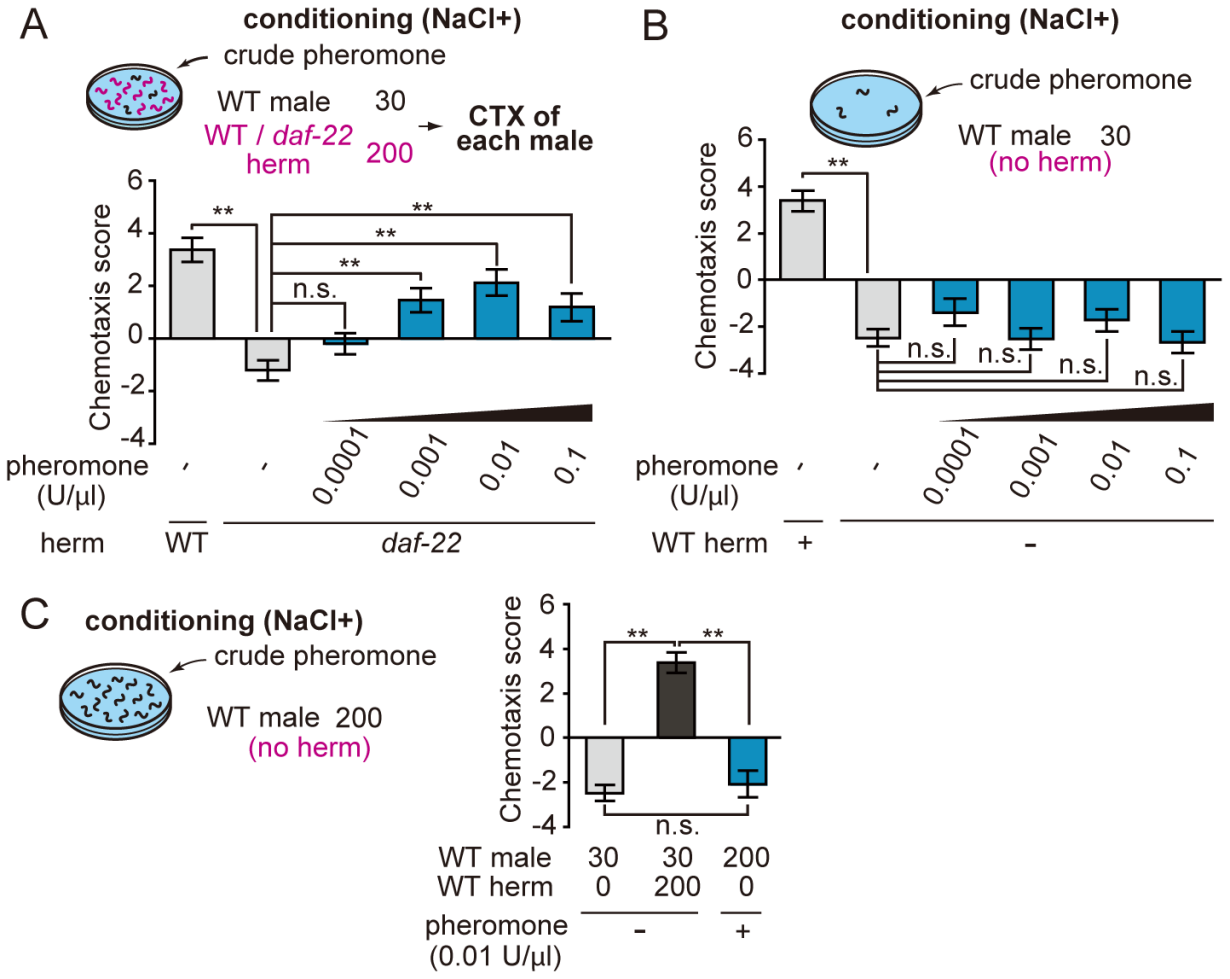
Pheromonal signaling is essential but not sufficient for sexual conditioning. (A) The *daf-22(m130)* hermaphrodite (*daf-22* herm) shows defects in sexual conditioning, which are rescued by addition of crude pheromone to the conditioning plate. Pheromone rescues sexual conditioning in *daf-22* hermaphrodites **, *P*<0.001; +, *P*<0.05; n.s., not significant. Error bars represent SEM. n = 18–36 animals. (B) Crude pheromone did not support sexual conditioning when applied without the *daf-22* hermaphrodite. (C) Sexual conditioning does not occur on the pheromone-containing plate with a high-density population of males. **, *P*<0.001; n.s., not significant. Error bars represent SEM. n = 38–63 animals for each condition.

In sexual conditioning, the effect of hermaphrodites antagonizes that of starvation (Fig. 2*A*), suggesting that males prioritize mate searching over food exploration. In order not to waste the cost of mate searching, it should be important for males to precisely discriminate hermaphrodites from males and distinguish the same species from closely related nematode species. We therefore asked how males recognize hermaphrodites to receive sexual signals. We first examined the involvement of pheromones in sexual conditioning. *C. elegans* continuously secretes the dauer pheromone, which includes a mixture of sugar derivatives called ascarosides [12], [25]. The pheromones regulate the developmental decision of dauer larva formation [29], and also attract males [9]–[11]. Composition of the pheromones are different between two sexes and male secretion is not attractive to males [9], [30]. We found that sexual conditioning requires *daf-22*, which encodes an enzyme in the pheromone biosynthesis pathway [25]: sexual conditioning did not occur when the WT male was conditioned with the *daf-22(m130)* hermaphrodites (Fig. 3*A*). In addition, sexual conditioning was restored by addition of crude pheromone extract into the agar plate on which the WT males were conditioned with salt and *daf-22* hermaphrodites (Fig. 3*A*), indicating that pheromones are essential for sexual conditioning.

### Pheromonal Signaling is not Sufficient for Sexual Conditioning

We next asked whether pheromones are sufficient to confer the hermaphrodite effect. Males were subjected to salt/starvation conditioning on plates supplemented with pheromones but without hermaphrodites, and subsequently single animals were tested for salt chemotaxis behavior. This procedure did not mimic the effect of sexual conditioning across the tested concentration range of pheromones (Fig. *3B*), suggesting that sexual conditioning requires additional sensory cue(s) not contained in crude pheromones but carried by the WT or *daf-22* mutant hermaphrodites. To investigate what might be acting as an additional cue(s), we asked whether the cue is specifically associated with hermaphrodites. We created a high density population consisting of only males and subjected them to salt/starvation conditioning on the plate supplemented with pheromones. If males also carry the second cue, addition of pheromones would restore sexual conditioning, as it did to *daf-22* hermaphrodites. However, sexual conditioning was not observed by this procedure (Fig. *3C*), suggesting that the second cue is hermaphrodite-specific. The second cue could be any compound secreted specifically from hermaphrodites but is lost during the pheromone extraction process, or components associated with the body surface structures of hermaphrodites but not males. Alternatively, the second cue may be transmitted through mating processes such as ejaculation.

### The Hermaphrodite Cuticle may Provide the Second Cue for Sexual Conditioning

**Figure 4:**
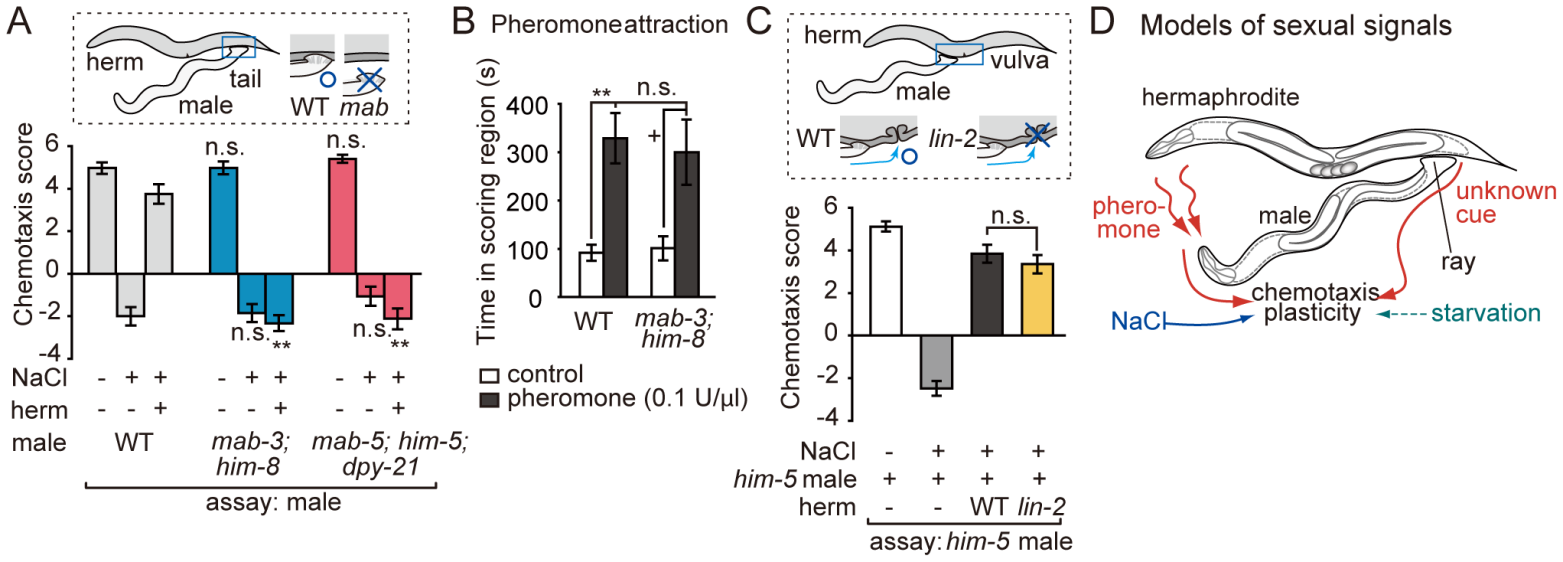
Sexual conditioning requires the male tail function. (A) The *mab-3(e1240); him-8(e1489)* and *mab-5(e1239); him-5(e1490); dpy-21(e428)* males were conditioned with the WT hermaphrodites. The *him-5* and *him-8* mutations were used to increase the incidence of males. The *mab-5(e1239); him-5(e1490); dpy-21(e428)* hermaphrodites produce *dpy* hermaphrodites and *non-dpy* males. These tail-defective males were defective in sexual conditioning. (B) Pheromone attraction assay (0.1 U/µl) of the *mab-3(e1240); him-5(e1490)* mutant. Tail-defective males respond normally to pheromones. (C) The WT males were conditioned with *lin-2(e1309)* vulvaless hermaphrodites. Vulvaless hermaphrodites can provide the cue for sexual conditioning. (D) Model for the sexual signals transmitted from hermaphrodites to males in sexual conditioning. The hermaphrodite pheromone and another cue are both required for sexual conditioning, and may be detected by the head and tale sensory systems of males, respectively. **, *P*<0.001; +, *P*<0.05; n.s., not significant. Error bars represent SEM. n = 49–69 animals for salt chemotaxis assay, and 16–27 animals for pheromone attraction assay.

When a male worm contacts a hermaphrodite, a male places its tail on the hermaphrodite body and searches for the vulva by moving its tail along the hermaphrodite body surface [15]. When the male locates the hermaphrodite vulva, it stops moving, inserts its spicules and ejaculates into the uterus. To assess the necessity of these mating processes for sexual conditioning, we examined mutants defective in mating behavior. Mutations of *mab-3* and *mab-5*, which encode a DM domain transcription factor and a homeodomain transcription factor, respectively, cause defects in the development of the ray sensilla, the sensory structures that contain nine pairs of sensory neurons in the male tail [31], [32] Due to this defect, these mutants cannot recognize the hermaphrodite body surface and do not mate. When the *mab-3(e1240); him-8(e1489)* and *mab-5(e1239); him-5(e1490); dpy-21(e428)* males were conditioned with the WT hermaphrodite, these mutants failed to receive sexual signals (Fig. 4*A*). Although the observed defect in sexual conditioning might be attributed to the defects of ray sensilla, another possibility is that the defect is due to defective perception of pheromones: *mab-3* is also expressed in the head neurons in addition to the tail neurons [33]. To test this possibility, we addressed whether the *mab-3* mutation affects pheromone perception in pheromone attraction assay. The *mab-3* mutant showed a normal phenotype in the pheromone attraction assay (Fig. 4*B*), suggesting that the unresponsiveness of *mab-3* to sexual conditioning is not due to the defect in pheromone perception, but likely to defects in spicule insertion.

As described earlier, the male mating behavior consists of tail contact, vulva location, spicule insertion and ejaculation [15]. All of these steps are affected by the *mab-3* and *mab-5* mutations. To narrow down the key steps for sexual conditioning, the vulvaless mutant *lin-2(e1309)* was tested for sexual conditioning. The *lin-2* gene is required for the induction of vulva and the *lin-2* hermaphrodite is devoid of vulva [34]. Thus, males are incapable of locating vulva or ejaculating into uterus of the *lin-2* hermaphrodites, but can only contact the body surface of the *lin-2* hermaphrodites. We found that when the *him-5(e1490)* males were conditioned with the *lin-2* hermaphrodites, the males received sexual signals normally (Fig. 4*C*), suggesting that the later steps of mating behavior are dispensable for the communication (Fig. 4*D*).

### The Neural Sex of Males is Important for Sexual Conditioning

**Figure 5:**
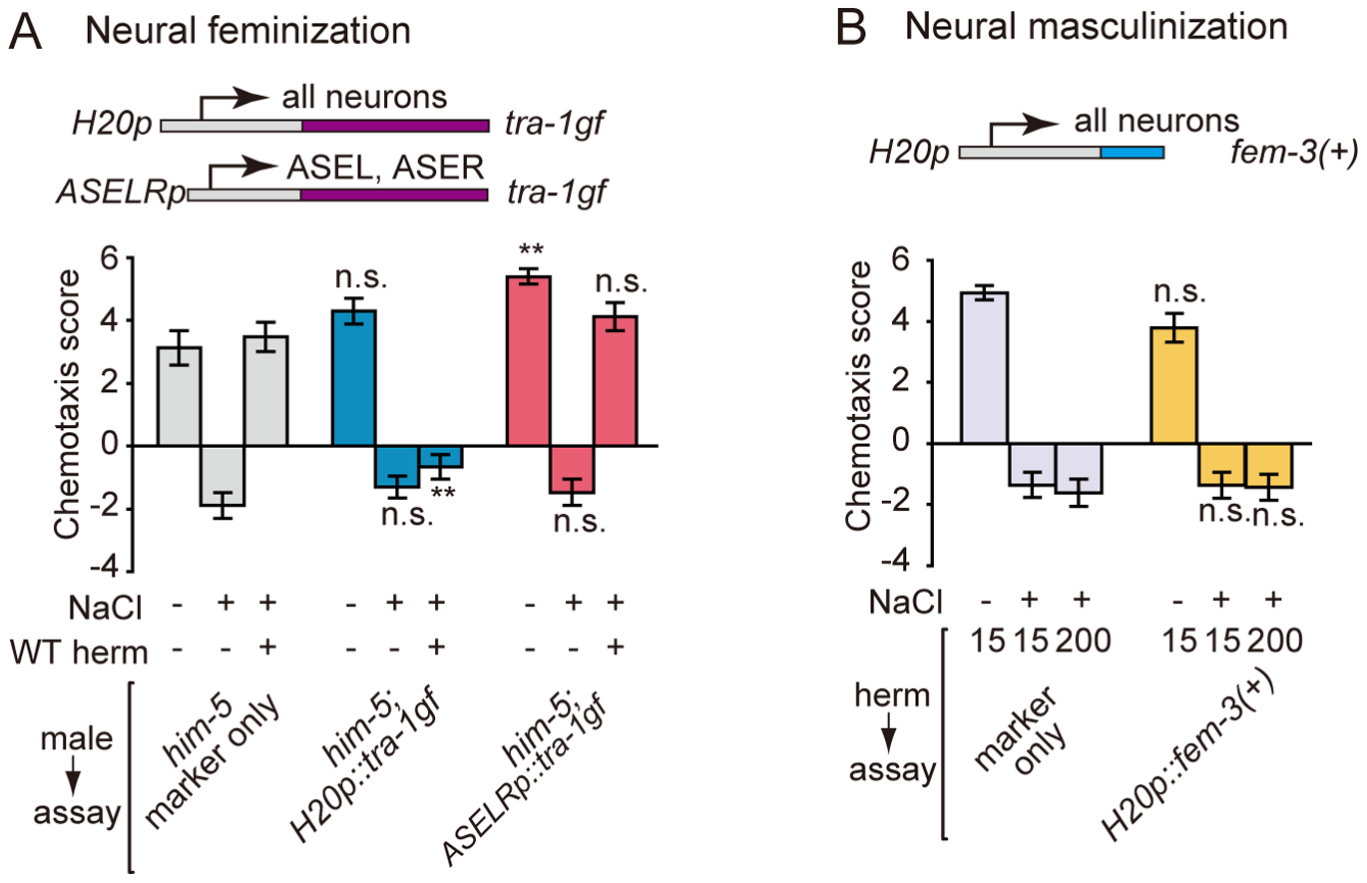
The impact of tissue- and cell-specific sex conversion on sexual conditioning. (A) *tra-1(gf)* was expressed in all neurons by the *H20* promoter or in ASE neurons by the *gcy-5* and *gcy-7* promoters to feminize the respective neurons of males, which were conditioned with the WT hermaphrodites. Males with feminized neurons are not attracted to NaCl after being conditioned with hermaphrodites. (B) Overexpression of *fem-3(+)* under the control of the *H20* promoter to masculinize all neurons. The transgenic hermaphrodites were conditioned in a high-density population, but this procedure does not cause sexual conditioning. **, *P*<0.001; n.s., not significant. Error bars represent SEM. n = 56–64 animals for each condition.

The male nervous system contains 87 male-specific neurons, most of which are located in the tail, in addition to the common set of neurons almost identical to hermaphrodites [6]. This architecture suggests that the male-specific behavior may be programmed by a simple addition of the male-specific peripheral sensory functions to the core behavioral program shared with hermaphrodites. Alternatively, or in addition, structurally similar neurons of the central neural circuits may be sexually diversified in functional aspects, such as connectivity and neurochemical features, thus producing sexual dimorphism of behavior [27]. To map the sites of sexual diversification for sexual conditioning, tissue- and cell-specific sex conversion experiments are feasible by manipulating sex determination genes *tra-1* and *fem-3* [27]. The zinc finger transcription factor TRA-1 controls essentially all somatic sexual dimorphisms in *C. elegans*, by repressing male fate and promoting hermaphrodite fate, whereas FEM-3 promotes male fate by negatively regulating TRA-1 35–37. First, we attempted feminization and masculinization of the whole nervous system. To feminize the nervous system of males, a gain-of-function form of TRA-1, TRA-1(N86D) [38], was expressed under the control of the pan-neuronal *H20* promoter. When this transgenic male was conditioned with the WT hermaphrodites, sexual conditioning failed to occur (Fig. 5*A*), suggesting that the sexual identity of the male nervous system is essential to receive sexual signals. To masculinize the whole nervous system of hermaphrodites, FEM-3(+) was expressed under the control of the *H20* promoter [27]. We subjected a high density population of these transgenic hermaphrodites to salt/starvation conditioning to ask whether the masculinized nervous system can receive sexual signals. The effect of sexual conditioning was not mimicked in these transgenic hermaphrodites (Fig. 5*B*). This result suggests that feminization of the core neural circuit in a hermaphrodite is not sufficient for generating male behaviors, and is consistent with the idea that the sexual conditioning requires the function of tail organ in males. In summary, the sexual identity of the neural tissues in the male is important for sexual conditioning.

Next we asked whether the ASE gustatory neurons undergo male-specific differentiation, because ASE neurons play a major role in sensing NaCl and are important regulatory sites in salt/starvation associative learning in previous studies [39]. We attempted cell-specific feminization of ASE by specifically expressing *tra-1(gf)* in the ASE neurons using the *gcy-5* and *gcy-7* promoters, which drive expression in the right and left member, respectively, of the morphologically symmetric ASE neuron pair. This transgenic male was conditioned with the WT hermaphrodites, and subsequently tested for salt chemotaxis behavior. In contrast to the feminization of the whole nervous system, cell-specific feminization of ASE did not cause defects in sexual conditioning (Fig. 5*A*). The site of sexual diversification may be peripheral neurons dedicated for receiving sexual information or neurons integrating sexual information and starvation signals.

## Discussion

### The Role of Sexually Conditioned Behavior in Mate Searching

We demonstrated that only males learn to associate salt taste with the presence of opposite sex. The sexual asymmetry in the behavior may be because males are not required for the reproduction of hermaphrodites, which can self-fertilize and therefore would naturally dedicate more resources to food exploration than mate search.

In the sexually conditioned males, the salt taste is associated with the presence of hermaphrodites. How does this association exert effects in the natural environment? The effect of sexual conditioning becomes manifest under starvation conditions, and a high density of hermaphrodites is required to fully antagonize the starvation effect (Fig. 2*A*). Although it is unlikely that hermaphrodites are attracted to places without food, it is possible that they form a high-density population under starvation conditions. The ecological cycle of *C. elegans* seems to consist of colonization to a bacterial food source, reproduction and dispersal from the colony. As reproduction proceeds, a growing population of worms forages on a decreasing bacterial food. When food is eventually exhausted, the population density reaches a peak and worms are crowded under starvation conditions. At this moment, the hermaphrodites are thought to quickly switch their overall sensory programs: they avoid the gustatory, olfactory and thermosensory cues associated with starvation, and switch their behaviors from foraging to dispersal [18]. Even before hermaphrodites actually starve, olfactory plasticity is promoted by the high population density, which is predictive of early food depletion [40]. In contrast to hermaphrodites, the high population density emerging around the moment of food depletion should be the best opportunity for males to mate with hermaphrodites. Even after the food supply is depleted, the males remain in the same colony until most of the hermaphrodites leave. This beneficial behavior is likely ensured by sexual conditioning, in which males positively associate the hermaphrodite presence with the environmental sensory cues. As hermaphrodites leave and the effect of starvation dominates, sexual conditioning no longer occurs and males begin to leave the colony. Thus sexual conditioning may set the dispersal order from a food-depleted colony: hermaphrodites leave first while males disperse later.

### Dual Sensory Cues Required for Sexual Conditioning

Our results suggest that sexual conditioning requires pheromonal signaling and at least one additional signal that is hermaphrodite-specific. Why does sexual conditioning require a combination of at least two sensory cues? As discussed above, sexual conditioning likely contributes to increasing mating opportunity at the cost of foraging opportunity. If males incorrectly recognize hermaphrodites, this mechanism costs both chances of survival and that of reproduction. Therefore sexual conditioning requires strict specificity for recognition of sex and species, and needs also to be timely. We suggest that these three requirements are secured by the dual sensory cues. First, sex specificity is likely ensured by pheromones: because males selectively respond to hermaphrodite-conditioned media, but not to male-conditioned media, sex-specific components might be contained in hermaphrodite secretion [9], [30]. Sex specificity also seems to be provided by another hermaphrodite-specific cue, because sexual conditioning did not occur on the pheromone-containing plate which did not contain hermaphrodites but contained a high density of males. Second, pheromones likely confer species specificity, but it may not be sufficient to fully ensure it: *C. elegans* males are attracted to female secretion of a dioecious species, *Caenorhabditis remanei* [41]. Although ecological segregation may prevent such interspecies attraction, it would be desirable that males can distinguish *C. elegans* hermaphrodites from related nematode species given the huge number of nematode species on earth. The second cue could be any non-pheromonal compound secreted specifically from hermaphrodites or components associated with the body surface structures of hermaphrodites but not males. This signal may perhaps complement distinction among related nematode species. Third, secreted pheromones report cumulative information for recent hermaphrodite presence, rather than real-time information, and therefore lacks timeliness. A high concentration of pheromones will remain well after hermaphrodite dispersal and, if pheromones alone were sufficient for sexual conditioning, males would be trapped in the food-depleted place where hermaphrodites are no longer present. We suggest that timeliness may be secured by direct contact of the male tail sensilla with hermaphrodite body surface, because tail-malformed males could not receive the sexual signal.

Although direct contact to hermaphrodite body surface was suggested to be essential in the regulation of dispersal strategy called “leaving behavior” [16], [17], the identity of the surface cue is also elusive for this behavior. When males search for the vulva by surveying the body surface of hermaphrodites, they often fail to locate the vulva or it takes time to locate it. Thus, body surface cue is easier to sense than possible vulval cues or ejaculation cues, and may therefore be important for sexual communication in nematode species. Whether the second cue used for sexual conditioning is in fact a hermaphrodite-specific body surface compound or other secreted molecules is an interesting subject of the future studies.

### Neural Circuits of Sexual Conditioning

The neural feminization experiment suggests that neural sex is essential to receive sexual signals in males. Then what part of the male nervous system is sexually differentiated and how are the sexual signals integrated with the starvation signal to switch gustatory behavior? Although, in principle, any step from sensory input to behavioral output may be altered to change salt chemotaxis behavior, the change is likely produced within a small circuit composed of ASE and its downstream interneurons because gustatory and olfactory plasticity are independently regulated [23]. Furthermore, growing evidence suggests that the gustatory switch from attraction to repulsion in salt/starvation conditioning is largely regulated in ASE [39], [42]–[44]. We postulated that, in a similar manner, sexual conditioning may directly or indirectly regulate the activity of ASE to change the direction of chemotaxis. The ASE-specific feminization experiment suggested that the sexual differentiation in ASE neurons is not important. In one possible model based on this piece of information, the sexual information may be integrated with starvation information at a point upstream of ASE, and this integrated information may be transmitted to ASE to modulate its salt-sensing functions. For example, the sexual information may block the activity of starvation-responsive neurons that direct starvation-induced aversive learning. A prediction of this model would be that other sensory modalities regulated by food availability, such as olfaction and thermosensation [18], may similarly be subject to sexual conditioning. Furthermore, the effects of food on other behaviors such as those on locomotory rate [45] may perhaps be mimicked by the hermaphrodite presence. The entire behavioral programs governed by sexual conditioning are issues of future studies.

Our results demonstrate a highly coordinated behavioral program in males. Indeed, at least four sensory stimuli including salt, starvation, pheromones and the possible body surface cue are integrated in sexual conditioning. In addition to the integration of starvation and sexual information as discussed in above models, it also remains to be determined where and how pheromones and the tail-derived signal are integrated. Previous studies described individual functions of head and tail sensory systems involved in sexual communication [9]–[11], [16], [17], and coordination of head and tail neurons that coordinate the mate recognition and appetitive drives [46]. Our findings add to this increasing knowledge on the tight coordination of male behaviors and provide another platform to explore the computational mechanisms for integrative behaviors.

## Supporting Information

Figure S1 **Assay formats for behavioral assays.** A) The assay format for salt chemotaxis assays. Each animal was given a score based on the sum of scores of the sectors through which the animal had traveled. B) The assay format for pheromone attraction assays. Two microlites of diluted pheromones and water were each spotted within one of two circles of 5 mm diameter, which are defined as a scoring region and a control region, respectively. The length of time animals spent in conditioned region was scored and was defined as “Time in scoring region”.
(TIF)

Figure S2 **Animals raised on OP50 bacterial lawns were also affected by sexual conditioning.** Animals raised on OP50 were subjected to the behavioral assay. Aversive learning is suppressed in the presence of hermaphrodites during salt/starvation conditioning as animals raised on NA22. **, P<0.001 Error bars represent SEM. n = 55–65 animals for each condition.
(TIF)
