# Supplementary figures and images for: A Sexually Conditioned Switch of Chemosensory Behavior in C. elegans
Source: PLoS One. 2013 Jul 4;8(7):e68676. doi: 10.1371/journal.pone.0068676 (PMC3701651; doi:10.1371/journal.pone.0068676)

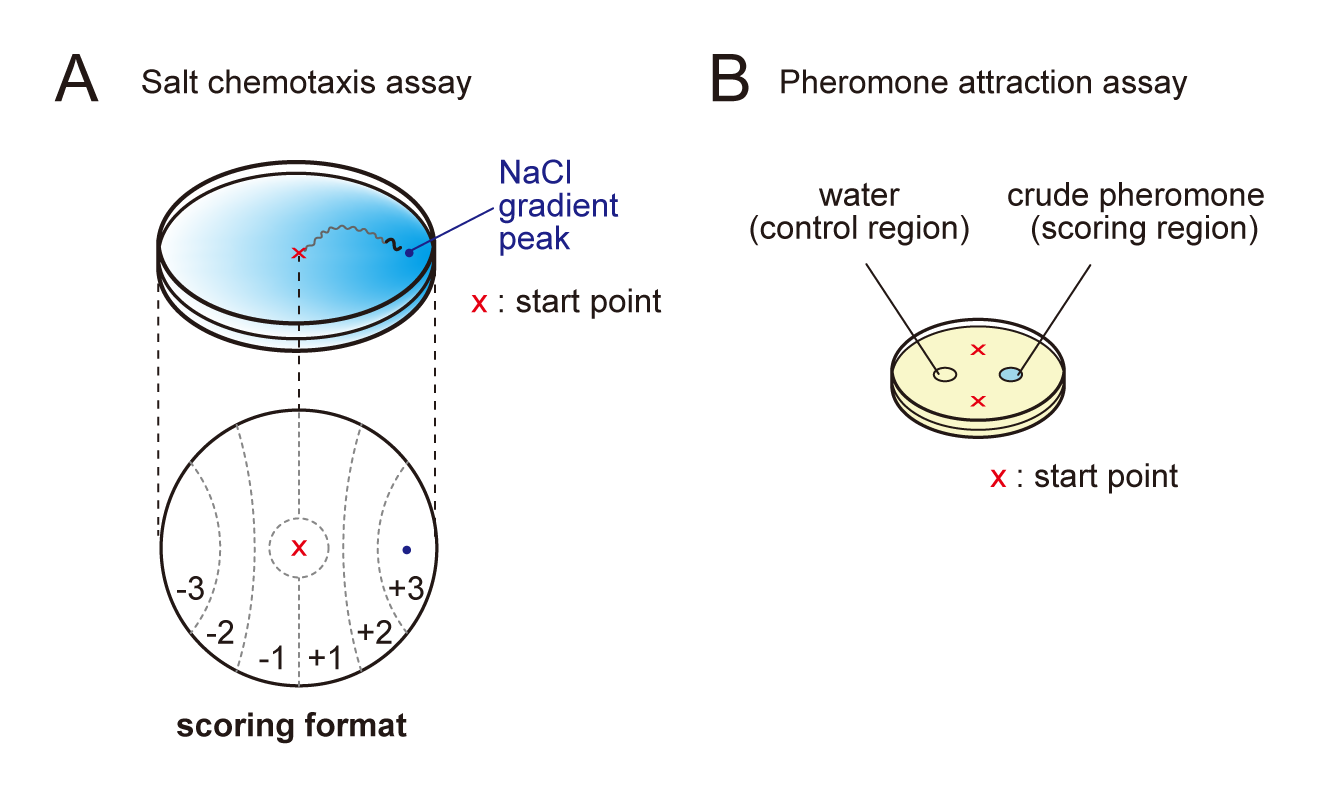

Supplement: Figure S1 — Assay formats for behavioral assays. A) The assay format for salt chemotaxis assays. Each animal was given a score based on the sum of scores of the sectors through which the animal had traveled. B) The assay format for pheromone attraction assays. Two microlites of diluted pheromones and water were each spotted within one of two circles of 5 mm diameter, which are defined as a scoring region and a control region, respectively. The length of time animals spent in conditioned region was scored and was defined as “Time in scoring region”. (TIF) [file pone.0068676.s001.tif]

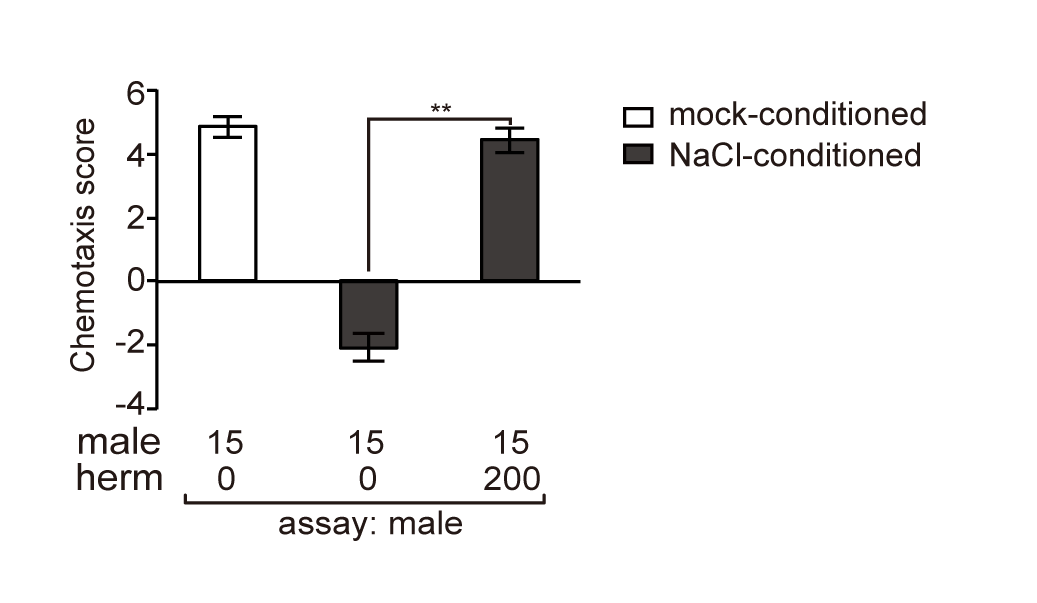

Supplement: Figure S2 — Animals raised on OP50 bacterial lawns were also affected by sexual conditioning. Animals raised on OP50 were subjected to the behavioral assay. Aversive learning is suppressed in the presence of hermaphrodites during salt/starvation conditioning as animals raised on NA22. **, P<0.001 Error bars represent SEM. n = 55–65 animals for each condition. (TIF) [file pone.0068676.s002.tif]
